# The Effect of Incomplete Bile Duct Obstruction on Diisopropanolnitrosamine—Induced Cholangiocarcinoma

**DOI:** 10.1155/1991/65315

**Published:** 1991

**Authors:** Yoshio Kinami, Yoshinao Ashida, Keitaro Seto, Shigeki Takashima, Ichiro Kita

**Affiliations:** 1 Department of Surgery II, and Division of Cancer Research Kanazawa Medical University Uchinada Ishikawa Japan; 2 Medical Research Institute Kanazawa Medical University Uchinada Ishikawa Japan; 3 Department of Surgery II Kanazawa Medical University Uchinada-Machi Kahoku-Gun, Ishikawa 920-02 Japan

## Abstract

This study was carried out to clarify the influence of incomplete bile duct obstruction (IBDO) on the occurrence and proliferation of cholangiocarcinoma and to evaluate the effect of release of IBDO at an early stage, using 175 Syrian golden hamsters. These hamsters received 500mg/kg body weight of diisopropanolnitrosamine (DIPN) once weekly for 10 weeks, and then were divided into 3 groups, consisting of the simple laparotomy group (SL group), the IBDO group and 2 week IBDO group, in which IBDO was released after 2 weeks. The occurrence rates of cholangiocarcinoma at 20 weeks were 42% in the SL group, 76% in the IBDO group and 30% in the 2 week IBDO group. The mean numbers of tumors per hamster in the IBDO group were significantly greater than those in other groups (p < 0.05). Both occurrence rates and numbers of tumors in the 2 week IBDO group were similar to those in the SL group. The proliferation of bile ductules and isolation of bacteria from bile in the IBDO group had higher rates at 15, 20 weeks than those found in the other groups. These results suggest that IBDO has an influence, as promoter, on the occurrence of cholangiocarcinoma induced by DIPN, and the disappearance of its promoting effect is caused by release of the obstruction.


*HPB Surgery*, 1991, Vol. 3, pp. 117–127
Reprints available directly from the publisher
Photocopying permitted by license only

© 1991 Harwood Academic Publishers GmbH
Printed in the United Kingdom

### THE EFFECT OF INCOMPLETE BILE DUCT OBSTRUCTION ON DIISOPROPANOLNITROSAMINE—INDUCED CHOLANGIOCARCINOMA

YOSHIO KINAMI[1,2], YOSHINAO ASHIDA[1], KEITARO SETO[1], SHIGEKI TAKASHIMA[1] and ICHIRO KITA[1]

*Department of Surgery II[1], and Division of Cancer Research, Medical Research Institute[2], Kanazawa Medical University, Uchinada, Ishikawa, Japan*

This study was carried out to clarify the influence of incomplete bile duct obstruction (IBDO) on the occurrence and proliferation of cholangiocarcinoma and to evaluate the effect of release of IBDO at an early stage, using 175 Syrian golden hamsters. These hamsters received 500mg/kg body weight of diisopropanolnitrosamine (DIPN) once weekly for 10 weeks, and then were divided into 3 groups, consisting of the simple laparotomy group (SL group), the IBDO group and 2 week IBDO group, in which IBDO was released after 2 weeks. The occurrence rates of cholangiocarcinoma at 20 weeks were 42% in the SL group, 76% in the IBDO group and 30% in the 2 week IBDO group. The mean numbers of tumors per hamster in the IBDO group were significantly greater than those in other groups ($p < 0.05$). Both occurrence rates and numbers of tumors in the 2 week IBDO group were similar to those in the SL group. The proliferation of bile ductules and isolation of bacteria from bile in the IBDO group had higher rates at 15, 20 weeks than those found in the other groups. These results suggest that IBDO has an influence, as promoter, on the occurrence of cholangiocarcinoma induced by DIPN, and the disappearance of its promoting effect is caused by release of the obstruction.

KEY WORDS: Cholangiocarcinoma, diisopropanolnitrosamine, incomplete bile duct obstruction, bile ductules, bile infection

### INTRODUCTION

The occurrence of cholangiocarcinoma in the liver has been observed during the follow-up of patients with hepato-biliary diseases accompanying morphological abnormality in the intra- or extrahepatic bile duct, and including intrahepatic stone disease[1–3]. Bile stasis and cholangitis caused by incomplete or complete bile duct obstruction are recognized commonly in these diseases. There is an inferred relationship between these clinical features and the occurrence of cholangiocarcinoma. Yamamoto[4] reported that in rats with obstruction of the choledochus, proliferation of bile ductules was an important factor in the occurrence of cholangiocarcinoma induced by diethylnitrosamine (DENA). Kinami *et al.*[5] administered diisopropanolnitrosamine (DIPN) to hamsters with incomplete bile duct obstruction (IBDO), and observed the promoting effect of IBDO on the occurrence of cholangiocarcinoma. These investigations reveal that obstruction of the bile duct is an important factor in the occurrence of cholangiocarcinoma, and simultaneously suggest its clinical significance in the release of bile duct obstruction at an early stage. This study was carried out to clarify experimentally these observations.

---

Address for correspondence: Dr Y.Kinami, Department of Surgery II, Kanazawa Medical University, Uchinada-Machi, Kahoku-Gun, Ishikawa 920-02, Japan.

### MATERIALS AND METHODS

One hundred and seventy male 8-week-old Syrian golden hamsters were used. These animals were kept under standardized conditions (room temperature, 23 ± 5°C; humidity, 60 ± 20%), and received MM-3 pellet diet (Funabashi Co.) and water ad libitum. As a carcinogen, 500mg/kg body weight (10ml/kg) of DIPN (Tokyo Chemical Co.) in saline was administered subcutaneously to all hamsters once weekly for 10 weeks. At 10 weeks, these hamsters underwent procedures as follows, and were divided into 3 groups. Forty hamsters underwent a simple laparotomy (SL) as a sham operation after being anesthetized by intraabdominal injection of 50 mg sodium pentobarbital/kg body weight, and were named the 'SL group'. Using the same anesthetic, 65 hamsters underwent IBDO by means of ligation of the choledochus adjacent to the pancreas, using 6-0 catgut. These were named the 'IBDO group'. Forty-five hamsters underwent IBDO by means of loose ligation of the choledochus in order to avoid complete obstruction, using 7-0 silk. After 2 weeks, the knot was untied and the silk removed to release the obstruction. These hamsteres were named the '2 week IBDO group'. Some hamsters dropped out due to various causes over the 20 week period, so that 36 of the 'SL group', 54 of the 'IBDO group' and 35 of the '2 week IBDO group' were evaluated. In addition, 6 hamsters before and another six hamsters 10 weeks after the beginning of DIPN administration were used to examine the serum bilirubin and alkaline phosphatase, or bacteria in the bile.

The hamsters in each group were sacrificed 12, 15 and 20 weeks after the beginning of DIPN administration, in order to perform the various examinations. After collecting the bile, a mixture of barium sulfate and gelatin was infused into the choledochus of 15 hamsters in each group, and the liver was removed and fixed in 10% formalin. Then, cholangiography was carried out using softex photography to investigate the condition of the bile duct. Thirty-six hamsters of the SL group, 54 of the IBDO group and 35 of the 2 week IBDO group were used to examine the histological changes in the liver. The liver fixed with formalin was sliced at 3 mm intervals. Each slice was embedded in paraffin, and sectioned at 7 $\mu$. The sections were stained with hematoxylin and eosin, and then examined under light microscopy. Histological changes in the intrahepatic bile duct, occurrence rates, number per hamster and maximum diameter of cholangiocarcinoma were studied in hamsters of each group.

The bilirubin and alkaline phosphatase levels in serum and the isolation rates of bacteria from bile were determined. The biochemical determination was performed in 18 hamsters of each group. The bacteriological examination was carried out in 25 hamsters of the SL group, 28 of the IBDO group and 27 of the 2 week IBDO group. Blood taken from the inferior vena cava was centrifuged for 15 min at 3000 rpm, and the serum was obtained. The determinations of bilirubin and alkaline phosphatase levels were performed using an autoanalyzer (736-type, Hitachi Co.). In the SL and 2 week IBDO group, a cannula was inserted into the choledochus, and the bile was collected by natural flow. In the IBDO group, the dilated choledochus was punctured, and the bile was absorbed. To detect bacteria in the bile, an EG agar plate (Eiken Chemical Co.) was used, and the culture was carried out at 37°C for 48 hours. A GasPak® system (BBL) was used to incubate anaerobes. The identification of bacteria was performed using gram stain and the vitek system (VITEK).

Statistical analysis was performed as follows. The values measured were presented as the mean ± SD. Scheffe's multiple comparison was adopted to compare the values among the groups, and Dunn's multiple comparison was used for comparison of the number of tumors. Cox's method was used for comparison of percentage data.

### RESULTS

### 1. The bile duct findings in cholangiograms

The cholangiograms at 12, 15 and 20 weeks in the IBDO group and at 12 weeks in the 2 week IBDO group exhibited marked dilatation of intra- and extrahepatic bile ducts and incomplete obstruction at the lower choledochus. The stricture, obstruction or displacement of the intrahepatic bile duct caused by invasion of tumors was observed in hamsters with occurrence of cholangiocarcinoma of the IBDO group at 20 weeks. But these changes were obscure in hamsters with tumor of the other groups. In the 2 week IBDO group, the cholangiograms at 15 and 20 weeks revealed the disappearance of bile duct dilatation and incomplete obstruction in the choledochus (Figure 1).

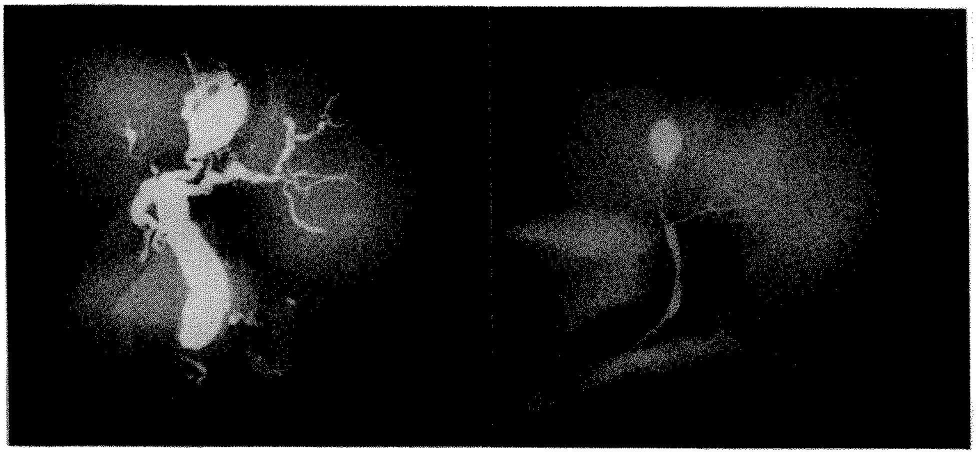

**Figure 1** Cholangiograms of hamsters in the IBDO group (a) and in the 2 week IBDO group (b) at 20 weeks. (a): showing marked dilatation and stenosis of the intra- and extrahepatic bile duct. (b): showing disappearance of marked dilatation.

### 2. Pathological findings in the liver

Tumors in the liver, appearing as white nodules of 0.1–1.7 cm in size, were observed in hamsters of all groups 15 and 20 weeks after beginning of DIPN administration. These tumors revealed cholangiocarcinoma associated with minute connective stroma in histological examination, in which carcinoma cells formed acini and trabeculae of various sizes (Figure 2 a). Invasion of carcinoma cells into the liver parenchyma was observed around the lesion.

Histological changes in the intrahepatic bile duct were seen in hamsters of each group. The proliferation of bile ductules, including cystic formation, goblet cell metaplasia and papillary hyperplasia of the bile duct epithelium were the main benign changes in the liver (Figure 2 b-d). The occurrence rates of proliferation of bile ductules or cystic bile ductules at 15 and 20 weeks in the IBDO group were higher than those in the other groups, but there was no significant difference (Table 1). The occurrence rates of goblet cell metaplasia or papillary hyperplasia in this group were also higher than those in the other groups, but there was no significant difference. In the 2 week IBDO group, the occurrrence rates of these histological changes after release of obstruction were similar to those in the SL group.

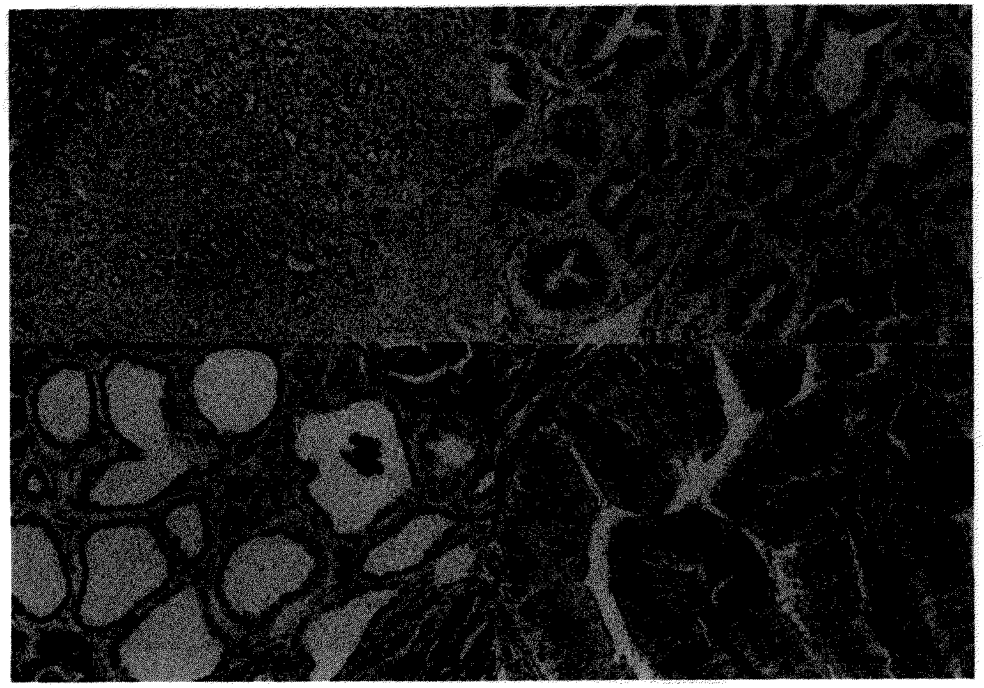

**Figure 2** Histological findings of the intrahepatic bile duct in hamsters with administration of DIPN. (a): cholangiocarcinoma associated with minute connective stroma. (HE, X 50). (b): proliferation of bile ductules. (c): proliferation of cystic bile ductules. (d): papillary hyperplasia. (HE, X 250).

3. Occurrence and proliferation of cholangiocarcinoma

The occurrence rates of cholangiocarcinoma at 15 and 20 weeks in the IBDO group were 64% and 76% respectively, and were higher than those in the other groups (Table 2). There was a significant difference in the occurrence rate at 15 weeks between the SL and IBDO groups ($p < 0.05$). The occurrence rates at respective weeks in the 2 week IBDO group were similar to those in the SL group.

**Table 1** Histological changes in the intrahepatic bile duct in each group

| | *Time after beginning of DIPN administration* | | | | | | | | | | | |
|---|---|---|---|---|---|---|---|---|---|---|---|---|
| | *12 weeks* | | | | *15 weeks* | | | | *20 weeks* | | | |
| *Groups* | *Ductules* | *Cystic ductules* | *Goblet*° *cell meta* | *Papillary hyperplasia* | *Ductules* | *Cystic ductules* | *Goblet*° *cell meta* | *Papillary hyperplasia* | *Ductules* | *Cystic ductules* | *Goblet*° *cell meta* | *Papillary hyper-plasia* |
| SL | 42% (5/12) | 8% (1/12) | 0% (0/12) | 8% (1/12) | 58% (7/12) | 67% (8/12) | 0% (0/12) | 25% (3/12) | 67% (8/12) | 92% (11/12) | 17% (2/12) | 33% (4/12) |
| IBDO | 55% (6/11) | 46% (5/11) | 0% (0/11) | 18% (2/11) | 77% (17/22) | 91% (20/22) | 27% (6/22) | 27% (6/22) | 95% (20/21) | 95% (20/21) | 38% (8/21) | 48% (10/21) |
| 2 week IBDO | 55% (6/11) | 46% (5/11) | 0% (0/11) | 18% (2/11) | 57% (8/14) | 86% (12/14) | 7% (1/14) | 29% (4/14) | 70% (7/10) | 90% (9/10) | 10% (1/10) | 30% (3/10) |

( ) Number of hamsters. ° Goblet cell metaplasia.

**Table 2** Occurrence rates for cholangiocarcinoma and number of tumors per hamster in each group

| | *Time after beginning of DIPN administration* | | | |
|---|---|---|---|---|
| | *15 weeks* | | *20 weeks* | |
| *Groups* | *Occurrence rates* | *Number of tumors*△ | *Occurrence rates* | *Number of tumors*△ |
| SL | 17% (2/12)[a] | 1.0±0[a] | 42% (5/12) | 1.2±0.4[a] |
| IBDO | 64% (14/22)[b] | 3.4±1.8[b] | 76% (16/21) | 3.2±1.7[b] |
| 2 week IBDO | 29% (4/14) | 1.0±0[c] | 30% (3/10) | 1.3±0.6[c] |

( ) Number of hamsters. △ Per hamster (mean ± SD). [a–b,b–c] $p<0.05$.

The mean numbers of tumors per hamster at 15 and 20 weeks in the IBDO group were significantly larger than those in the other groups ($p < 0.05$), whereas there was no difference between the SL and 2 week IBDO groups. Regarding the tumor size, tumors in hamsters with IBDO were slightly larger than those in hamsters without it, but there was no significant difference (Table 3).

**Table 3** Relationship between the size and number of tumors in each group

| | *Time after beginning of DIPN administration* | | | | | |
|---|---|---|---|---|---|---|
| | *15 weeks* | | | *20 weeks* | | |
| | *Size of tumors (mm)* | | | *Size of tumors (mm)* | | |
| *Groups* | *–5* | *5–10* | *10–* | *–5* | *5–10* | *10–* |
| SL | 100% (2) | 0% | 0% | 67% (4) | 33% (2) | 0% |
| IBDO | 70% (35) | 22% (11) | 8% (4) | 41% (21) | 22% (11) | 37% (19) |
| 2 week IBDO | 75% (3) | 25% (1) | 0% | 25% (1) | 50% (2) | 25%(1) |

( ) Number of tumors.

4. Changes in serum bilirubin and alkaline phosphatase

As shown in Table 4, the serum bilirubin levels in the SL group were constant over a 20 week period, while those in the other groups were elevated after incomplete obstruction of the choledochus. In the 2 week IBDO group, the levels were reduced following release of obstruction. The serum alkaline phosphatase levels in all groups were elevated after beginning of DIPN administration. The levels in the SL group were constant 10 week later, but those in the IBDO group were elevated still further. The levels in the 2 week IBDO group were reduced following release of obstruction, and were similar to those in the SL group at 20 weeks.

5. Isolation rates of bacteria from bile

Bacteria were not detected in the bile of the SL group throughout the whole course, but were detected in the bile of 2 groups receiving IBDO after 12 weeks (Table 5). The isolation rates of bacteria from bile at 15 and 20 weeks in the IBDO

group were 82% and 70%, respectively and were higher than those in the 2 week IBDO group. Except for unidentified bacteria, those of 13 varieties, including E. coli, Bacteroides, Streptococcus and others, were isolated from bile of 2 groups with IBDO.

**Table 4** Changes of serum bilirubin and alkaline phosphatase (AL-P) levels in each group

| | | | | *After beginning of DIPN administration* | | | |
|---|---|---|---|---|---|---|---|
| *Groups* | *Examinations* | *n* | *Before* | *10 weeks* | *12 weeks* | *15 weeks* | *20 weeks* |
| SL | | 6 | 0.3±0.1 | 0.5±0.2 | 0.4±0.2[a] | 0.4±0.2[c] | 0.4±0.2[c] |
| IBDO | Bilirubin levels○ | 6 | 0.3±0.1 | 0.5±0.2△ | 3.4±2.0[b] | 5.9±2.5[d] | 5.1±0.7[d] |
| 2 week IBDO | | 6 | 0.3±0.1 | 0.5±0.2△ | 3.4±2.0▲[b] | 0.5±0.2[e] | 0.5±0.2[e] |
| SL | | 6 | 146±19 | 310±38 | 340±38 | 314±22[c] | 298±20[c] |
| IBDO | AL-P levels○ | 6 | 146±19 | 310±38△ | 384±53 | 422±72[d] | 482±28[d] |
| 2 week IBDO | | 6 | 146±19 | 310±38△ | 384±53▲ | 376±91 | 316±42[e] |

○— mean ± SD. △ Incomplete obstruction. ▲ Release of obstruction. [a–b] $p<0.05$. [c–d, d–e] $p<0.001$.

**Table 5** Isolation rates of bacteria from bile in each group

| | *Isolation rates of bacteria* | | | | |
|---|---|---|---|---|---|
| | | *After beginning of DIPN administration* | | | |
| *Groups* | *Before* | *10 weeks* | *12 weeks* | *15 weeks* | *20 weeks* |
| SL | 0% (0/6) | 0% (0/6) | 0% (0/5) | 0% (0/12)[a] | 0% (0/8)[a] |
| IBDO | 0% (0/6) | 0% (0/6)△ | 29% (2/7) | 82% (9/11)[b] | 70% (7/10)[b] |
| 2 week IBDO | 0% (0/6) | 0% (0/6)△ | 29% (2/7)▲ | 30% (3/10) | 30% (3/10) |

( ) Number of hamsters. △ Incomplete obstruction. ▲ Release of obstruction. [a–b] $P<0.05$

### DISCUSSION

Carcinogens for inducing cholangiocarcinoma in animals,[1,6,7,8] are already well known. The DIPN used in this study is a substance discovered by Althoff *et al.*[9] and Pour *et al.*[10,11], who examined the carcinogenic effect of di-n-propylnitrosamine and its $\beta$-oxidized compounds in the Syrian golden hamster. They observed the occurrence of, not only pancreatic carcinoma, but also cholangiocarcinoma by DIPN administration[12]. Kinami *et al.*[5] studied the influence of IBDO on the occurrence of cholangiocarcinoma induced by DIPN in a previous experiment using hamsters, in which administration of the carcinogen was continued for 20 weeks and was started simultaneously with IBDO. We reported the promoting effect of IBDO on carcinogenesis. In this study the IBDO was performed after DIPN administration for 10 weeks, and its influence on the occurrence of cholangiocarcinoma was examined. Also, the effect of the release of obstruction at an early stage on carcinogenesis was also evaluated.

Ligation of the choledochus, using a silk or nylon thread, was generally performed as a means of bile duct obstruction[4]. The single ligation method of the choledochus using 6-0 catgut, making it possible to maintain the IBDO for a long term, and a loose ligation method using 7-0 silk were used in this study. The latter method performed in the 2 week IBDO group was beneficial because of the 2 weeks long maintenance of the IBDO and removal of the ligated silk. Cholangiography proved that these methods were suitable for the purpose of this study.

Pour *et al.*[12] observed the occurrence of hemangioendothelioma, angiosarcoma and hepatoma, in addition to cholangioma and cholangiocarcinoma, in the liver of hamsters administered 2, 2′-dihydroxy-di-n-propylnitrosamine dissolved in olive oil. In a similar examination, Kajikawa *et al.*[14] reported a hamster in which not only adenoma and cholangiocarcinoma, but also hemangioendothelioma was induced. On the contrary, our results revealed no other neoplasm, except proliferation of bile ductules and cholangiocarcinoma, in the liver of hamsters receiving DIPN dissolved in saline. Yamamoto[4] mentioned that the lesion of bile ductules proliferation acted as an important factor in the occurrence of cholangiocarcinoma in rats treated with DENA. In our examination on hamsters with DIPN, it was assumed that the changes in the epithelium of proliferated ductules were related to the occurrence of cholangiocarcinoma, because an increased number of bile ductules were seen around carinoma lesions. On the other hand, although the ductule proliferation and changes in the bile duct epithelium were mainly based on DIPN administration, our results indicated that the IBDO had a promoting effect on these pathological changes; these changes in the IBDO group occurred at higher rates than those in other groups. On the other hand, the occurrence rates of these pathological changes in the 2 week IBDO group after release of obstruction were similar to those in the SL group. Accordingly, this finding revealed that the effect of release of the obstruction could be seen histologically.

The occurrence rates for cholangiocarcinoma at 15 and 20 weeks in the IBDO group were higher than those in the SL and 2 week IBDO group; besides, these rates were higher than those seen in the literature[11,13]. The numbers of tumors per hamster in the IBDO group were significantly large, compared with those in other groups. Regarding the tumor size, tumors in the IBDO group were larger than those in other groups, but there was no significant difference. These findings show the promoting effect of IBDO on the occurrence and proliferation of cholangiocarcinoma and the absence of its effect in hamsters with IBDO for a short term.

As seen in the SL group, serum bilirubin levels did not change throughout the whole course, but serum alkaline phosphatase levels were elevated after beginning DIPN administration. It is presumed that the latter changes are caused by the carcinogen, and are related to the histological changes in intrahepatic bile ducts. Both levels were elevated after IBDO, but were reduced following its release. It is inferred that bacterial infection occurs in the bile with stasis caused by bile duct obstruction, and then, the conversion from primary to secondary bile acid is promoted[14,15]. However, our examination[5] performed previously did not reveal an increase in secondary bile acid in the infected bile of hamsters with IBDO. Phinney *et al.*[1] reported a case of cholangiocarcinoma associated with Caroli's disease, and stated that, with the onset of cholangitis, bacterial enzymes might become available to activate procarcinogens to the ultimate level of carcinogens. Regarding the results of culture in this study, the IBDO group, with high occurrence rates of tumor, exhibited high isolation rates of bacteria from bile. Besides, it was recognized that the release of bile duct obstruction at an early stage reduced bacterial infection in the bile, as seen in the results of the 2 week IBDO group.

### *References*

1. Phinney, P.R., Austin, G.E. and Kadell, B.M. (1981) Cholangiocarcinoma arising in Caroli's disease. *Arch.Pathol.Lab.Med.*, **105**, 194–197
2. Sanes, S. and MacCallum, J.D. (1942) Primary carcinoma of the liver, cholangioma in hepatolithiasis. *Am.J.Pathol.*, **18**, 675–687
3. Kinami, Y., Noto, H., Miyazaki, I. and Matsubara, F. (1978) A study of hepatolithiasis associated with cholangiocarcinoma. *Acta Hepatologica Japonica*, **19**, 578–583
4. Yamamoto, S. (1985) Experimental studies on the cause of cholangiocarcinoma—with special reference to obstructive lesions of bile ducts—. *Ochanomizu Medical J.*, **33**, 223–235
5. Kinami, Y., Ashida, Y. and Seto, K. (1988) Incomplete bile duct obstruction and cholangiocarcinoma induced by diisopropanolnitrosamine. *Gastroenterological Surgery*, **11**, 1831
6. Argus, M.F. and Hoch-Ligeti, C. (1961) Comparative study of the carcinogenic activity of nitrosamine. *J.Natl.Cancer Inst.*, **27**, 695–709
7. Weisburger, E.K., Ward, J.M. and Brown, C.A. (1974) Dibenamine: selective protection against diethylnitrosamine-induced hepatic carcinogenesis but not oral, pharyngeal and esophageal carcinogenesis. *Toxicol.Appl.Pharmacol.*, **28**, 477–484
8. Herrold, K.M. (1967) Histogenesis of malignant liver tumors induced by dimethylnitrosamine, an experimental study in Syrian hamsters. *J.Natl.Cancer Inst.*, **39**, 1099–1111
9. Althoff, J., Krueger, F.W. and Mohr, U. (1973) Brief communication: carcinogenic effect of dipropylnitrosamine and compounds related by $\beta$-oxidation. *J.Natl.Cancer Inst.*, **51**, 287–288
10. Pour, P., Krüger, F.W., Chem, D., Althoff, J., Cardesa, A. and Mohr, U. (1974) Cancer of the pancreas induced in the Syrian golden hamster. *Am.J.Pathol.*, **76**, 349–358
11. Pour, P., Krüger, F.W., Althoff, J., Cardesa, A. and Mohr, U. (1975) A new approach for induction of pancreatic neoplasms. *Cancer Research*, **35**, 2259–2268
12. Pour, P., Krüger, F.W., Althoff, J., Cardesa, A. and Mohr, U. (1975) Effect of beta-oxidized nitrosamines on Syrian hamsters. III. 2, 2′-dihydroxy-di-n-propylnitrosamine. *J.Natl.Cancer Inst.*, **54**, 141–146
13. Kajikawa, M., Nakazawa, S., Imai, K., Naito, Y., Ishikawa, M. and Kimoto, E. (1978) DHPN a study of experimental cholangiocarcinoma induced by in golden hamsters. *Igakunoayumi*, **105**, 74–76
14. Midtvedt, T. (1974) Microbial bile acid transformation. *Am.J.Clin.Nutr.*, **36**, 1341–1347
15. Ozawa, A. and Tazume, S. (1983) Intestinal flora and bile acids metabolism. *Saishin-Igaku*, **38**, 2410–2417

*(Accepted by S. Bengmark 22 March 1990)*

### INVITED COMMENTARY

Cholangiocarcinoma occurs throughout the biliary tree. As a result, the spectrum of this malignancy includes a purely intrahepatic variety, perihilar tumors (the so-called Klatskin tumor), distal bile duct tumors, and a diffuse intra- and extrahepatic variety. Of these various types, only the distal bile duct tumors have a moderate five-year survival of 30% to 50% following resection. Many of the intrahepatic cholangiocarcinomas remain silent initially and present as large, often unresectable and/or metastatic, liver tumors. The perihilar tumors that presumably present early with jaundice and are said to be slow-growing also have a poor prognosis. In most series 15% to 30% of patients with perihilar cholangiocarcinoma have peritoneal and/or liver metastases at the time of presentation. Moreover, even among the resectable perihilar tumors, reported five-year survival rates are only 10% to 25%.

The prognosis for cholangiocarcinoma remains poor, in part, because it is a rare entity. As a result, clinical studies to determine the pathogenesis and the results of therapy have been limited. For these reasons, a good animal model for cholangiocarcinoma is clearly needed. Thus, the present report by Kinami and his associates is a welcome addition to the literature. The model they are using, diisopropanolnitrosamine administration to the Syrian golden hamster, was first described 15 years ago but was then infrequently applied until the present author's 1988 report. This model should be appropriate to determine the effects of radiotherapy, various chemotherapeutic agents, and hormones on the growth of cholangiocarcinoma. To study the effect of these potential therapies, however, survival data will have to be determined.

Cholangiocarcinoma is known to occur with increased frequency in clinical situations associated with prolonged biliary stasis. These entities include Caroli's disease, choledochal cysts, primary sclerosing cholangitis, and intrahepatic stone disease. One pathogenetic theory has proposed that prolonged stasis increases exposure of the biliary epithelium to carcinogens that are excreted in bile. The present report by Kinami and his associates supports this theory. Measurements of diisopropanolnitrosamine or its metabolites in bile would be an interesting additional study. Further clinical evidence that carcinogens may be excreted in bile, or that bile may be a cocarcinogen, is the fact that periampullary tumors occur with increased frequency in the duodenum just distal to the ampulla.

The authors have clearly demonstrated that partial bile duct obstruction enhances the growth of cholangiocarcinoma in their model. Whether this enhanced tumor growth is the result of altered host immunity, increased concentration of the chemical carcinogen, a cocarcinogen, or some other effect remains unclear. Bile duct obstruction is known to alter hepatic reticuloendothelial, polymorphonuclear, and lymphocyte function. Obstructive jaundice is also associated with increased portal and systemic levels of endotoxin. This endotoxemia, in turn, may affect hepatic, renal, cardiac, and pulmonary function. These profound effects on the host may be sufficient to promote the growth of these animal, as well as human, cholangiocarcinomas.

The most important observation made by Kinami *et al.* is that if biliary obstruction is released after only two weeks, tumor growth is returned to baseline levels. This finding needs to be further explored to determine whether a critical period exists beyond which the effects of obstruction cannot be reversed. The reversible effect of biliary obstruction on tumor growth in this model is also of interest because of the lack of effect of relief of obstruction in clinical studies of operative morbidity and mortality. These differing results may be explained, however, by differences in the effects of 1) internal versus external biliary drainage, 2) partial versus complete obstruction, and 3) short versus long periods of obstruction.

Another interesting question raised, but not answered, by this study is whether bacterial infection of the biliary system also plays a procarcinogenic effect. Each of the clinical situations associated with prolonged bile stasis and an increased incidence of cholangiocarcinoma are also associated with bactibilia and recurrent episodes of cholangitis. In the present study the incidence of bactibilia closely paralleled the occurrence of cholangiocarcinoma. Interesting further studies in which the incidence and growth of cholangiocarcinoma could be monitored would explore the effect of 1) introducing bacteria into the biliary system at the time of partial bile duct ligation or 2) treating the animals with antibiotics. Again, the authors should be congratulated for reintroducing this animal model of cholangiocarcinoma to the hepato-pancreato-biliary literature.

Henry A. Pitt, M.D.
Professor of Surgery
The Johns Hopkins Medical Institutions
Baltimore, Maryland, USA

